# Supplementary material for: Effects of Organic Biostimulants Added with Zeolite on Zucchini Squash Plants Infected by Tomato Leaf Curl New Delhi Virus
Source: Viruses. 2022 Mar 15;14(3):607. doi: 10.3390/v14030607 (PMC8952782; doi:10.3390/v14030607)
Supplement: Supplementary file 1 [file viruses-14-00607-s001.zip › S1 Table of assignations of zucchini extracts.pdf]

Table S1. Peak Assignment of Metabolites Identified on Zucchini extracts by ^{1}H-NMR

Compound [a]	Assignment [b]	^{1}H δ (ppm)	Multiplicity[c]	^{13}C δ (ppm)
**Organic acids**				
Acetic acid (AA)	CH_3	1.92	**s**	25.98
Citric acid (CA)	α,γ-CH	2.67	d	44.77
	α',γ'-CH	2.71	d	44.77
**Chlorogenic acid (CGA)**	CH_2-2'	2.02,2.17	m	40.11
	CH-3'	5.33	m	40.14
	CH-4'	3.88	dd	75.43
	CH-5'	4.23	m	72.93
	**CH-8**	**6.39**	**d**	73.82
	CH-7	6.94	d	117.56
	CH-2	7.12	dd	118.90
	CH-5	7.19	d	117.81
	CH-6	7.65	d	149.59
**Formic acid (FA)**	**CH**	**8.46**	**s**	171.90
Fumaric acid (FumA)	CH=CH	6.51	s	137.94
Lactic acid (LA)	CH_3	1.32	d	22.95
	CH	4.11	q	77.04
**Malic acid (MA)**	**α- CH**	**4.31**	**dd**	69.33
	β.β'- CH	2.38,2.69	dd	40.86
**Neochlorogenic acid (nCGA)**	CH_2-2'	1.92,2.09	m	40.11
	CH-3'	4.04	dd	75.43
	CH-4'	3.61	dd	72.93
	CH-5'	5.35	m	43.23
	CH_2-6'	2.09,2.23	m	40.14
	**CH-8**	**6.43**	**d**	115.95
	CH-7	7.66	d	117.56
	CH-2	7.22	d	118.90
	CH-5	6.96	dd	117.81

	CH-6	6.15	d	149.59
Succinic acid (SA)	2 CH_2	2.39	s	36.31
**Amino acids**				
**Alanine (Ala)**	**β-CH_3**<br>α- CH	**1.49**<br>3.80	**d**<br>q	19.05<br>53.56
**Arginine (Arg)**	γ- CH_2<br>β- CH_2<br>**δ- CH_2**<br>α- CH_2	1.69<br>1.95<br>**3.21**<br>3.78	m<br>m<br>**t**<br>t	30.51<br>43.27<br>43.12<br>57.32
**Aspartic acid (Asp)**	**β'-CH**<br>β-CH<br>α-CH	**2.68**<br>2.72<br>3.91	**dd**<br>dd<br>m	39.31<br>39.31<br>55.09
**Asparagine (Asn)**	β'-CH<br>**β-CH**<br>α-CH	2.86<br>**2.89**<br>4.01	dd<br>**dd**<br>m	37.44<br>37.44<br>54.09
**Glutamate (Glu)**	γ- CH_2<br>**β,β-CH_2**<br>α-CH	2.07<br>**2.35**<br>3.78	m<br>**m**<br>m	29.25<br>37.12<br>58.01
**Glutamine (Gln)**	γ- CH_2<br>**β,β-CH_2**<br>α-CH	2.11<br>**2.45**<br>3.81	m<br>**m**<br>m	29.31<br>34.02<br>57.19
**Histidine (His)**	CH-6<br>CH-7<br>CH-5<br>**CH-2**	3.19<br>3.99<br>7.09<br>**8.01**	dd<br>dd<br>d<br>**d**	30.25<br>57.29<br>120.03<br>138.31
**Isoleucine (Ile)**	δ-CH_3<br>**γ-CH_3**<br>γ'-CH<br>γ''-CH<br>β-CH<br>α-CH	0.95<br>**1.02**<br>1.25<br>1.49<br>1.99<br>3.69	t<br>**d**<br>m<br>m<br>m<br>m	13.85<br>17.38<br>27.01<br>27.01<br>38.71<br>63.04
**Leucine (Leu)**	**δ,δ'- CH_3**	**0.97**	**m**	23.85, 24.59

	γ-CH<br>β-CH_2<br>α-CH	1.72<br>1.73<br>3.74	m<br>m<br>m	26.81<br>42.60<br>56.21
**γ-aminobutyric acid (GABA)**	**β-CH_2**<br>γ-CH_2<br>α-CH_2	**1.95**<br>2.30<br>3.01	**t**<br>m<br>t	26.38<br>37.06<br>42.21
**Phenylalanine (Phe)**	CH-2,6<br>CH-4<br>**CH-3,5**<br>β-CH_2<br>α-CH	7.32<br>7.38<br>**7.42**<br>3.27<br>3.98	d<br>d<br>**d**<br>m<br>dd	130.3<br>128.6<br>130.3<br>37.1<br>56.8
**Threonine (Thr)**	**γ-CH_3**<br>α-CH<br>β-CH	**1.33**<br>3.60<br>4.27	**d**<br>m<br>m	22.15<br>63.46<br>68.94
**Tyrosine (Tyr)**	CH-2,6<br>**CH-3,5**<br>β-CH_2<br>α-CH	7.22<br>**7.08**<br>3.15<br>3.93	d<br>**d**<br>dd<br>dd	130.0<br>117.0<br>37.1<br>56.8
**Valine (Val)**	γ-CH_3<br>**γ'-CH_3**<br>β-CH<br>α-CH	0.99<br>**1.05**<br>2.29<br>3.62	d<br>**d**<br>m<br>m	19.41<br>20.75<br>31.89<br>63.36
**Carbohydrates**				
**α-Glucose (α-G)**	**CH-1**<br>CH-2<br>CH-3<br>CH-4<br>CH-5<br>CH_2-6	**5.25**<br>3.55<br>3.72<br>3.42<br>3.84<br>3.73, 3.90	**d**<br>m<br>m<br>m<br>m<br>m	93.10<br>72.49<br>73.84<br>70.67<br>72.52<br>96.97
**β-Glucose (β-G)**	**CH-1**<br>CH-2<br>CH-3<br>CH-4	**4.69**<br>3.26<br>3.50<br>3.42	**d**<br>m<br>m<br>m	96.97<br>75.17<br>76.84<br>70.70

	CH-5	3.48	m	74.57
	CH_2-6	3.74, 3.91	m	61.80
**α-Xylose (α-X)**	**CH-1**	**5.17**	d	93.1
	CH-6	3.80	m	72.5
	CH-3	3.70	m	73.8
	CH-5	3.45	m	70.7
	CH-2	3.35	m	72.5
	CH_2-6	3.73-3.90	m	96.9
**β-Xylose (β-X)**	**CH-1**	**4.56**	d	97.0
	CH-5	3.87	m	74.6
	CH-3	3.69	m	76.8
	CH-4	3.36	m	70.7
	CH-2	3.18	m	75.2
**Sucrose (S)**	**GLC CH-1**	**5.42**	**d**	93.22
	CH-2	3.59	m	72.11
	CH-3	3.79	m	73.54
	CH-4	3.48	m	70.26
	CH-5	3.85	m	73.38
	CH_2-6	3.82	m	61.18
	FRU CH_2-1’	3.69	m	62.44
	C-2	\	\	104.85
	CH-3’	4.22	m	77.45
	CH-4’	4.06	m	75.04
	CH-5’	3.90	m	82.44
	CH_2-6	3.82	m	63.38
**Raffinose (R)**	CH-1 Glucose	5.45	d	96.41
	CH-2 G	3.55	dd	73.69
	CH-3 G	3.74	dd	75.34
	CH-4 G	3.53	dd	72.05
	CH-5 G	4.05	m	74.11
	CH2-6 G	4.69-4.02	m	68.37
	**CH-1 Galactose**	**5.01**	**d**	101.08
	CH-2 g	3.82	dd	71.03
	CH-3 g	3.89	dd	72.04

	CH-4 g	3.98	dd	83.88
	CH-5 g	3.95	m	73.71
	CH2-6 g	3.73	m	63.72
	CH-1 Fructose	3.67	s	64.01
	C-2 F	/	/	104.88
	CH-3 F	4.22	d	79.00
	CH-4	4.05	dd	79.69
	CH-5	3.88	m	83.98
	CH2-6	3.77-3.82	m	65.05
**Lipids & Sterols**				
**Stearic acid (SFA)**	CH_3	0.87	t	14.05
	n-CH_2	1.26	m	29.32
	C<u>H</u>$_2$-CH_2-CO_2^-	1.62	m	24.61
	**CH_2-CO_2^-**	**2.30**	**t**	33.52
**Linoleic acid (ω-6)**	CH_3	0.86	t	14.06
	n-CH_2	1.36	m	29.37
	C<u>H</u>$_2$-CH=CH	2.04	m	29.45
	CH=CH	5.37	m	130.29; 128.45
	**=CH-C<u>H</u>$_2$-CH=**	**2.76**	**t**	25.68
	C<u>H</u>$_2$-CH_2-CO_2^-	2.06	m	24.75
	CH_2-CO_2^-	2.31	t	34.05
**β-Sitosterol (β-ST)**	CH_2-1	1.08, 1.85	m	37.19
	CH_2-2	1.51, 1.84	m	31.50
	CHOH-3	3.52	m	71.81
	CH_2-4	2.28	m	42.37
	CH-6	5.34	m	121.79
	CH_2-7	1.52, 1.98	m	31.98
	CH-8	1.46	m	31.78
	CH-14	0.99	m	56.74
	CH_2-15	1.57	m	24.25
	CH_2-16	1.26, 1.85	m	28.37
	**CH_3-18**	**0.68**	**s**	12.20
	CH_3-25	1.01	s	19.12
**Campsterol (Camp)**	CH_2-1	1.08, 1.85	m	37.19

	CH_2-2	1.51, 1.84	m	31.50
	CHOH-3	3.52	m	71.81
	CH_2-4	2.28	m	42.37
	CH-6	5.34	m	121.79
	CH_2-7	1.52, 1.98	m	31.98
	CH-8	1.46	m	31.78
	CH-14	0.99	m	56.74
	CH_2-15	1.57	m	24.25
	CH_2-16	1.26, 1.85	m	28.37
	**CH_3-18**	**0.70**	**s**	12.21
	CH_3-25	1.01	s	19.12
**Miscellaneous Metabolites**				
**Choline (Chn)**	**$N(CH_3)_3$**	**3.20**	**s**	56.70
**Betaine (Bet)**	**N-$(CH_3)_3$**<br>CH_2	**3.26**<br>3.84	**s**<br>s	55.95<br>68.58
**Trigonelline (Trg)**	N-CH_3	4.42	s	51.1
	CH4	8.07	m	130.4
	CH3,5	8.82	m	148.5
	**CH1**	**9.11**	**s**	148.1
**Uracile (Ur)**	**CH-5**	**5.92**	**d**	103.7
	CH-6	7.85	d	146.3
**Nicotinamide adenine dinucleotide (NAD)**	**CH-2**	**9.45**	**s**	151.42
	CH-4	9.21	dd	153.25
	CH-6	8.95	dd	138.89
	CH-5	8.48	m	126.84
**Monoacylglycerol (MAG)**	**CH_2**	**3.65-3,55**	**dd**	65.45
	CH_2	4.05-4.15	dd	70.32
	CH	3.82	m	75.12
**Phospholipids (PP)**	CH	**5.13-5.21**	bm	77.45
	**2CH_2**	4.15-4.29	dd	68.23
**Carotenoids (Crt)**	CH_2-2,2’	1.47	m	39.62
	CH_2-3,3’	1.62	m	19.27
	CH_2-4,4’	2.02	m	33.18
	CH-7,7’	6.15	d	126.68

	CH-8,8’	6.14	d	137.78
	CH-10,10’	6.14	d	130.88
	**CH-11,11’**	**6.68**	**m**	125.04
	CH-12,12’	6.35	d	137.26
	CH-14,14’	6.25	d	132.45
	CH-15,15’	6.63	m	130.02
	CH_3-16,16’,17,17’	1.03	s	29.01
	CH_3-18,18’	1.72	s	21.77
	CH_3-19,19’	1.97	s	12.81
**Total Chlorofylls**	CH-5	9.57	s	137.6
	CH-10	9.22	s	108.2
	**CH-20**	**8.41**	**s**	93.4

In bold are evidenced the resonances chosen for metabolite quantification; s: singlet, d: doublet, t: triplet, q: quadruplet, dd: doublet of doublets, m: multiplet, bm: broad multiplet.
